# Project Panopia: cost-effective model for glaucoma referral refinement from community optometrists without the need for repeat testing

**DOI:** 10.1038/s41433-020-01133-1

**Published:** 2020-08-24

**Authors:** Rashmi G. Mathew, Connor J. Beddow, Hayley Raison, Dawn A. Sim

**Affiliations:** Moorfields Eye Hospital NHSFT, London, EC1V 2PD UK

**Keywords:** Health care economics, Optic nerve diseases

## The context

The quality and productivity gains that we seek lie at the heart of primary and secondary healthcare interface [1]. In the context of glaucoma, this involves referrals from community optometrists where there is currently a 40% false-positive rate (England, UK) [2, 3]. Multiple reports have shown that referral refinement has the potential to work [4–6]. One refinement scheme demonstrated up to 89% of glaucoma referrals could be kept out of Hospital Eye Service [HES] when Humphrey visual fields, Goldmann applanation tonometry and disc imaging were included [6]. Despite this, the cost-effectiveness of such schemes remains to be proven, largely due to the requirement of repeat testing within such pathways.

With the success of referral refinement programmes over the past two decades, why have these programmes been unable to scale? Interoperability of software platforms is a key barrier at the interface of primary and secondary care, and a priority area for the National Health Service (NHS) England [7]. Healthcare interoperability is hindered by data stored on heterogeneous proprietary legacy systems, which prevents interoperability with other vendor applications and protects market share [8].

## Ideals, intervention and causal assumptions

### Ideals

Telemedicine advocates the conceptualisation of the desired future state first, before finding the right technology to suit the workflow [9]. We extended this ideal to managing glaucoma by creating a two-way feedback mechanism between primary and secondary care and providing a one-stop service for patients close to home, with resultant improved access to secondary care within a cost-effective pathway.

### Intervention

A direct glaucoma pathway was set up between community optometrists and HES in order to provide a remote review service. The pathway bypassed current barriers of interoperability by utilising ‘nhs.net’ mail as an interim technology to facilitate the transfer of information. Requisite to the pathway was the provision of complete datasets by community optometrists. We called our model Panopia, to symbolise a concept where we take ‘a whole view of the referral pathway’.

### Causal assumptions

The causal assumptions underpinning the intervention being that remote oversight of referrals would reduce false positives to HES without the need for repeat testing, and consistent feedback would improve the quality of the referrals.

## Methods

A feasibility study utilising the new pathway was undertaken with 20 referrals. The primary aims, secondary aims and remote decisions of the feasibility study are listed in Table 1. Prior to the commencement of Panopia, we engaged stakeholders, which included Newham clinical commissioning group [CCG], local general practitioners and the local optometrists. An enhanced optometric tariff of £46 for each referral was agreed.

**Table 1:** (A) Outlines the primary aims of the Panopia feasibility study. (B) Outlines the secondary aims of Panopia feasibility study and results.

A. Primary aims	Onboarding experience (*n* = 23)
To assess the feasibility of recruitment and retention of optometry practices	•Eighteen local practices welcomed the scheme •Six optometric practices committed to the feasibility study and were our early adopters •Twelve practices could not participate in the initial phase due heavy administrative burden from the newly introduced MECS scheme •None of the practices dropped out during the course of the study
To assess the acceptability of the pathway among optometrists	•CET-accredited onboarding events were utilised to present the status quo and gather optometrists views on the intervention •Delays into HES and high first visit discharge rates were identified as a key motivator for change by optometrists •Additional time required to export and upload images to nhs.net email was not expressed as a concern and could be done during allocated administrative time •The referral proforma was co-created with optometrists
To investigate pre-existing resources within the community that would facilitate the pathway	•There was a heterogeneity of equipment in all six practices •Two of six optometric practices had OCT capabilities and a decision was made to utilise optic disc photographs instead •Only practices with visual field machines *and* fundus cameras were able to participate •Visual fields from any type of field analyser were accepted •Although GAT was initially specified, we also accepted NCT measurements
To assess the feasibility of data transfer and interpretation	•All practices were able to export data from their VF machines and fundus cameras and upload them on to nhs.net email •All practices utilised a bespoke referral proforma, which served as a checklist to ensure all information was provided in the referral •Data interpretation was possible on all referrals

To evaluate financial projections for scaling up					
Current scheme			Panopia scheme		
Based on 853 referrals to Moorfields at Barking Glaucoma clinic in a year	Costs £	Notes	Based on 853 referrals to Moorfields at Barking Glaucoma clinic in a year	Costs £	Notes
Community Optom costs	17,913	853 referrals × £21	Community Optom costs	39,238	853 referrals × £46
Additional optometry training and equipment costs	0		Additional optometry training and equipment costs	0	
HES first visit cost	150,981	853 referrals × £177	HES virtual review	102,360	853 referrals × £120 HES Virtual tariff (68% of multiprofessional first visit cost)
HES follow-up visit cost	34,494	665 referrals (78% that would not be referred to HES if Panopia utilised) ×0.57 × £91 1.57 extra visits prior to discharge from HES (1 New + 0.57 F/U) £91 = F/U tariff	HES follow-up visit cost	17,108	188 referrals seen in HES × £91
Total	203,388		Total	158,706	
Cost saving for 853 patients £203,388–158,706 = £44,682					
Cost saving per patient £44,682/853 = £52					
NB: 1.57 visits prior to discharge is for all ophthalmology outpatients and is taken from the Manchester glaucoma Enhanced referral scheme [14]					
NB: If we calculate costs based on 1.0 visits prior to discharge, rather than 1.57 visits per discharge, the scheme still makes a saving of £12 per patient					
The figure for HES virtual review quoted as £120 represents 68% of an F2F first visit appointment and is a baseline figure suggested on the NHSE/I tariff release for 2019;, this figure would need to be agreed at a local level and in the context of any local arrangements such as block contracting to ensure that providers are appropriately remunerated for this high volume work. This still allows a saving at the CCG level while ensuring any reduction in ‘new F2F activity’ in a HES setting does not impact negatively on the secondary care providers					
We anticipate that reduced F2F activity would allow improved RTT and access to service, meaning that HES would never be below its functional capacity					

B. Secondary aims			
To assess whether remote decisions can be made on new glaucoma referrals with a complete dataset from referring optometrists	Remote decisions could be made on all referrals.		
	Provisional diagnosis, *n* = 23	Referral outcome, *n* = 23	Referral to HES, *n* = 5
	Glaucoma suspect (suspicious disc, normal IOP, normal VF) 43% (10)	Discharge 26% (6)	Referral time frame::urgent (<2 weeks)/soon (2–4 weeks)/routine (<12 weeks)
	Narrow angles 17% (4)	Annual review with optom 35% (8)	
	Primary open-angle glaucoma 13% (3)	(annual review with optom 17% (4)—narrow angles)	Urgent: 4% (1 pt.)
	Normal tension glaucoma 4% (1)	HES review 22% (5)	Soon: 17% (4 pts)
	Primary angle closure 4% (1)		Routine: 0% (0 pts)
	Ocular hypertension 4% (1)		
	Non-glaucomatous disc 4% (1)		
	No abnormality detected 9% (2)		
To set up a Learning Network and embed learning into the pathway, to facilitate upskilling of optometrists	•Onboarding events included practical skills training for GAT, VH, disc assessment •All optometrists received referral advice and feedback within 5 days •One to two referrals were chosen monthly as an educational case/s with annotated disc images and VF communicated via email to all optometrists		

*MECS* minor eye conditions, *CET* continuing education and training, *HES* hospital eye service, *OCT* optical coherence tomography, *GAT* Goldmann applanation tonometry, *VF* visual field, *VH* Van Herick limbal chamber depth, *IOP* intraocular pressure, *CCG* Clinical commissioning group, *F2F* face to face,* RTT* referral to treatment

A complete dataset in an optometry referral included demographic details, reason for referral, family history of glaucoma, visual acuity, refraction, intraocular pressure (non-contact tonometry [NCT] or Goldmann applanation tonometry), Van Herick limbal chamber depth grade [VH], images of disc photographs, images of visual fields (any field type). A bespoke referral proforma was used. Optic disc photos and visual fields were transferred using nhs.net email. NHS.net email is automatically encrypted and complies with pan-government secure email standard and is therefore suitable for sharing patient sensitive information.

Feedback on each referral was sent to the referring optometrist within 5 days using nhs.net email. This work was registered with the Service Improvement Department of Moorfields Eye Hospital and complies with the criteria defined in the Declaration of Helsinki.

## Comment

Panopia was launched with a view to improve the current glaucoma referral model and to symbolise a concept whereby ‘a whole view of the new glaucoma patient pathway’ is taken.

Like any incremental innovation, feasibility studies are important to assess the processes involved and anticipate unforeseen difficulties. In this paper, we outline the key steps in order to initiate a new pathway, the change management required for implementation and undertook financial modelling to assess cost-effectiveness. As with any complex intervention, tailoring of the scheme was needed from the outset to suit the local context [10].

In this feasibility study, we demonstrated that we were able to deliver this scheme with high fidelity to the originally intended proposal. A total of 23 new glaucoma referrals were received between July and December 2019. The mean age of the cohort was 49 ± 13 years (range: 20–69). Forty-five per cent were female. Mean best-corrected visual acuity was −0.02 RE and −0.01 LE, and mean intraocular pressure was 18 mmHg (range 9–39 mmHg). A provisional diagnosis was made on 100% of referrals (see Table 1), with the most common reason for referral being suspicious optic discs (43%), highlighting the importance of disc imaging. Between 61 and 78% of referrals could be kept out of HES, depending on the risk threshold set.

Utilisation of existing optometry equipment without repeat testing was unique to the model and represented a paradigm shift in glaucoma referral refinement pathways. Equipment heterogeneity and ingrained use of NCT were important considerations, and decision making based on community-acquired data has been historically hindered by these factors. Integral to the scheme and decision making was the provision of a complete dataset by the optometrist for remote assessment by an ophthalmologist in HES.

‘Repeat measures’, ‘enhanced case finding’ and ‘referral refinement’ filtration schemes have already been in existence for more than two decades and often involve a second visit for the patient to repeat tests with standard equipment. In 1997, an audit of false-positive glaucoma referrals to HES, found they were discharged after 2.3 visits [11, 12]. A figure of over two visits has been incorporated into financial modelling for many referral filtering schemes [4, 12, 13]. It has since been refined in 2019 by the Manchester Glaucoma Enhanced referral scheme, which observed that the figure was reduced to 1.57 HES visits prior to discharge for all ophthalmic subspecialities [14], challenging the cost-effectiveness of existing filtering schemes. Using this more conservative estimate, the cost savings of our model were £52 (1.57 HES visits) and £12 per patient (1.0 HES visits) (see Table 1).

Another unique feature of the scheme is the Panopia Learning Network, which engaged individual optometrists with personalised feedback, such as annotations on disc images and bidirectional dialogue, with an end goal of improving referral quality.

Patient experience and safety must be at the heart of any healthcare change. Panopia is a proof of concept that this model provides a vehicle for expedited diagnosis, communication with patients and review in HES. It further reduces the anxiety associated with long waits (5 months from the time of optometry referral to diagnosis, R.G. Mathew, personal communication) and has the potential to improve patient safety from delayed appointments [15].

Panopia was conceived with the vision for a future model of glaucoma care. We applied an incremental change to enable stakeholder acceptance of both new pathways and technology. Human–technology interactions are likely to play a key role in the success of such pathways, and feasibility studies like these are important for scalability. In our scheme up to 78% of referrals could be kept out of HES by acquiring a minimum dataset transferred via secure email and with a cost saving of £52 per patient. Embracing technology that automates data acquisition and a transfer would allow scaling of this model and boost ‘out-of-hospital’ care as envisaged by the NHS Long-Term Plan [16].

## Summary

### What was known before

- There is at least a 40% false-positive rate for glaucoma referrals seen in HES.
- This delays appointments for those with true pathology.
- Current filtration schemes show variable cost-effectiveness.

### What this study adds

- This pilot of a remote review of glaucoma referrals with complete datasets from community optometrists showed that 78% of referrals could be kept out of HES, with a potential cost saving of £52 per patient.

### How might these results influence clinical practice

- Scaling up of this model with technology that automates data acquisition and transfer would significantly boost ‘out-of-hospital’ care.

## References

[1] Nicholson D. Implementing the next stage review visions: the quality and productivity challenge. Letter 10 August 2009, Gateway ref: 12396.

[2] Bowling B, Chen SD, Salmon JF (2005). Outcomes of referrals by community optometrists to a hospital glaucoma service. Br J Ophthalmol.

[3] Founti P, Topouzis F, Hollo G, Cvenkel B, Iester M, Haidich A (2018). Prospective study of glaucoma referrals across Europe: are we using resources wisely?. Br J Ophthalmol.

[4] Ratnarajan G, Newsom W, French K, Kean J, Chang L, Parker M (2013). The impact of glaucoma referral refinement criteria on referral to, and first-visit discharge rates from, the Hospital Eye Service: The Health Innovation & Education Cluster (HIEC) Glaucoma Pathways Project. Ophthalmic Physiol Opt.

[5] Bourne RRA, French KA, Chang L, Borman AD, Hingorani M, Newsom WD (2010). Can a community optometrist-based referral refinement scheme reduce false-positive glaucoma hospital referrals without compromising quality of care? The Community and Hospital Allied Network Glaucoma Evaluation Scheme (CHANGES). Eye.

[6] Trikha S, Macgregor C, Jeffery M, Kirwan J (2012). The Portsmouth-based glaucoma refinement scheme: a role for virtual clinics in the future?. Eye.

[7] Heubusch K (2006). Interoperability: what it means, why it matters. J AHIMA.

[8] Olaronke I, Oluwaseun O. Big data in healthcare: Prospects, challenges and resolutions. 2016 Future Technologies Conference (FTC), San Francisco, CA; 2016. pp. 1152–57. 10.1109/FTC.2016.7821747.

[9] Saleem SM, Pasquale LR, Sidoti PA, Tsai JC. Virtual ophthalmology: telemedicine in a Covid-19 era. Am J Ophthalmol. 2020. 10.1016/j.ajo.2020.04.029. 10.1016/j.ajo.2020.04.029 PMC7191296 32360862

[10] Orsmond GI, Cohn ES (2015). The distinctive features of a feasibility study: objectives and guiding questions. OTJR.

[11] Henson DB, Spencer AF, Harper R, Cadman EJ (2003). Community refinement of glaucoma referrals. Eye.

[12] Devarajan N, Williams GS, Hopes M, O’Sullivan D, Jones D (2011). The Carmarthenshire Glaucoma Referral Refinement Scheme, a safe and efficient screening service. Eye.

[13] Parkins DJ, Edgar DF (2011). Comparison of the effectiveness of two enhanced glaucoma referral schemes. Ophthalmic Physiol Opt.

[14] Forbes H, Sutton M, Edgare DF (2019). Impact of the Manchester Glaucoma Enhanced Referral Scheme on NHS costs. BMJ Open Ophthalmol.

[15] Foot B, MacEwen C (2017). Surveillance of sight loss due to delay in ophthalmic treatment or review: frequency, cause and outcome. Eye.

[16] NHS England. NHS Long Term Plan. 2019. https://www.longtermplan.nhs.uk/. Accessed 22 Jun 2020.

